# Clinical efficacy of transjugular intrahepatic portosystemic shunt in treating esophageal variceal bleeding in patients with liver cirrhosis

**DOI:** 10.12669/pjms.41.4.11695

**Published:** 2025-04

**Authors:** Qun Zhao, Qiong Zi, Guoqiang Dong, Yong Lu, Yingzhan Zhang

**Affiliations:** 1 Qun Zhao Department of Interventional Medicine, The Second Affiliated Hospital of Bengbu Medical College, Bengbu, Anhui Province 233040, P.R. China; 2 Qiong Zi Department of Urology, The Second Affiliated Hospital of Bengbu Medical College, Bengbu, Anhui Province 233040, P.R. China; 3 Guoqiang Dong Department of Interventional Medicine, The Second Affiliated Hospital of Bengbu Medical College, Bengbu, Anhui Province 233040, P.R. China; 4 Yong Lu Department of Interventional Medicine, The Second Affiliated Hospital of Bengbu Medical College, Bengbu, Anhui Province 233040, P.R. China; 5 Yingzhan Zhang Department of Interventional Medicine, The Second Affiliated Hospital of Bengbu Medical College, Bengbu, Anhui Province 233040, P.R. China

**Keywords:** Esophageal variceal bleeding, Liver cirrhosis, Somatostatin, Transjugular intrahepatic portosystemic shunt

## Abstract

**Objective:**

To explore the clinical efficacy of transjugular intrahepatic portal vein shunt (TIPS) in treating esophageal variceal bleeding (EVB) of liver cirrhosis patients.

**Methods:**

Clinical data of 60 patients with liver cirrhosis and EVB, admitted to The Second Affiliated Hospital of Bengbu Medical College between from November 2017 to April 2024, were retrospectively analyzed. Of them, 31 received TIPS treatment (TIPS group), and 29 received treatment with somatostatin (SST group). Blood transfusion volume, hemostasis time, and the length of hospital stay were compared between the two groups. Hemodynamic status, gastric motility indicators, vascular endothelial function indicators, levels of total bilirubin (TBIL), albumin (ALB), and international normalized ratio (INR) of both groups were compared before and after the treatment. The rebleeding and prognosis of patients were assessed after 6 months of follow-up.

**Results:**

The blood transfusion volume, hemostasis time, and length of hospital stay were significantly lower in the TIPS group than in the SST group (*P*<0.05). After the treatment, the hemodynamic status, gastric motility indicators, and vascular endothelial function indicators, in both groups significantly improved compared to those before treatment and were considerably better in patients treated by TIPS group compared to the SST group (*P*<0.05). There were no significant changes in the pretreatment and post-treatment levels of TBIL, ALB, and INR between the two groups (*P*>0.05). After six months of follow-up, the TIPS group showed significantly lower re-bleeding and mortality rates than the SST group (*P*<0.05).

**Conclusions:**

Compared with conventional drug therapy, TIPS treatment can more effectively regulate hemodynamic status, improve gastric motility and vascular endothelial function, and reduce re-bleeding and mortality of patients with liver cirrhosis complicated by EVB.

## INTRODUCTION

Cirrhosis is a common chronic liver disease that manifests as fibrotic replacement of liver tissue that can result from any chronic hepatic disease.^1^ Early liver cirrhosis lacks specific clinical manifestations. As the disease progresses, liver dysfunction and portal hypertension can lead to ascites portal hypertension and other complications.^2^ Esophageal variceal bleeding (EVB), caused by the rupture of submucosal distal esophageal veins that become dilated due to portal hypertension, is a serious complication of liver cirrhosis with a mortality rate of up to 14–35%.^3^-^5^ Timely implementation of safe and effective treatment for liver cirrhosis and EVB is crucial to ensure good patient outcomes.

Transjugular intrahepatic portal vein shunt (TIPS) is a commonly used method in the treatment of liver cirrhosis complicated by EVB.^6^ TIPS mainly relies on X-ray fluoroscopy induction to dilate the liver parenchyma between the hepatic and portal veins, place a stent, and construct a new pathway.^6^ Using metal stents to open new artificial blood vessels and to divert portal vein blood flow can reduce portal hypertension, control the degree of portal hypertension, and promote ascites absorption.^6^ This study aimed to retrospectively analyze the clinical data of 60 patients with liver cirrhosis complicated by EVB in order to explore the therapeutic effects of TIPS and provide a practical reference and evidence for the clinical treatment of the disease.

## METHODS

Clinical records of 60 patients with liver cirrhosis and EVB treated in The Second Affiliated Hospital of Bengbu Medical College from November 2017 to April 2024 were retrospectively selected. Patients were grouped according to their treatment methods and the treatment methods were selected by surgeon’s choice based on the condition of the patients.. Patients receiving TIPS comprised the TIPS group, and patients receiving somatostatin therapy (SST) were included in the SST group.

### Ethical Approval:

All procedures performed in the study involving human participants were in accordance with the ethical standards of the institutional and/or national research committee(s) and the Helsinki Declaration (as revised in 2013). The requirement for informed consent was waived by the ethics committee because of the observational and retrospective nature of the study. This study was approved by the Ethics Review Board of the Second Affiliated Hospital of Bengbu Medical College (No 2023233, Dated: 2023-12-23).

### Inclusion criteria:

- Met the diagnostic criteria for liver cirrhosis and EVB.^3^
- Diagnosis confirmed through gastroscopy examination.
- Aged ≥ 18 years.
- Complete clinical data.

### Exclusion criteria:

- Patients with digestive tract tumors.
- Patients who underwent other vascular interventions or surgical treatments during the study period.
- Patients with a history of endoscopic treatment, surgical diversion, or splenectomy
- Patients with coagulation dysfunction.
- Patients who received treatment with antiplatelet drugs, anticoagulants, and β receptor blockers within one month before surgery.

After the admission, all patients underwent routine interventions, such as acid suppression, hemostasis, and fluid replacement.

### SST method:

Patients received treatment with somatostatin: intravenous injection (0.1 g, followed by intravenous infusion of 25 μg/h.

### TIPS:

Preoperative routine blood ammonia, coagulation function, electrolytes, liver and kidney function levels, and blood routine examinations were done. Abdominal plain scan and enhanced CT examination were performed to clarify the liver and vascular conditions. The state of esophageal and gastric varicose veins were evaluated by gastroscopy. Patients were assisted in lying flat on the digital subtraction angiography operating table, and routine monitoring of finger pulse oxygen, blood pressure, and electrocardiogram were performed. Analgesics and sedatives were administered before performing the operation. The skin in the neck and groin area was disinfected and draped, local anesthesia was performed, and the right femoral vein and femoral artery were punctured.

A sheath tube and a pressure-measuring catheter were inserted to detect right atrial pressure. A catheter was introduced into the hepatic vein for labeling processing. Another catheter was inserted into the superior mesenteric artery for indirect anteroposterior and lateral portal vein angiography to clarify the position and morphology of the portal vein. Under local anesthesia, a right internal jugular vein puncture was performed using a COOK company puncture system (Rösch-Uchida Transjugular Liver Access Set, RUPS-100). Portal vein puncture was performed with the assistance of X-ray fluoroscopy, and a catheter was introduced after the puncture was successful. Portal vein angiography and pressure measurement were done, targeting the varicose veins presented in the angiography.

The spring coil or tissue glue embolus was selected according to the specific conditions, and the polyurethane foam was activated. An 8 mm Viatorr stent was used for inserting and measuring pressure through balloon dilation to determine the decrease in portal vein pressure gradient. Portal vein angiography was performed to evaluate the degree of stent patency. All sheaths and catheters were removed after the surgery, and the puncture site was compressed and bandaged. Close monitoring of finger pulse oxygen, blood pressure, and electrocardiogram status was performed within 24 hours after the surgery. Routine symptomatic support included infection prevention, anticoagulation, regulation of ammonia metabolism, and liver protection.

### Collected information:

- Baseline data, including sex, age, and liver cirrhosis grade and type.
- Hemodynamic status, including portal vein diameter (PVD), portal vein velocity (PVV), portal vein pressure (PVP), and portal vein flow (PVF). The above indicators were detected using a color Doppler ultrasound instrument (model: AI 5200s, American Donier company).
- Gastric motility indicators, including gastric antral contraction frequency (ACF), motility index (MI), and anstral contraction amplitude (ACA), were measured using a color Doppler ultrasound instrument (model: AI 5200s, American Donier Company).
- Vascular endothelial function indicators, including levels of endothelin (ET), nitric oxide (NO), and vascular endothelial growth factor (VEGF); 4ml of fasting venous blood was extracted and centrifuged at a radius of 5cm at 3000 r/min for five minutes. ET was measured by radioimmunoassay, levels of NO were assessed by the nitrate reductase method, and levels of VEGF were measured using the enzyme method, with the reagent kits purchased from Shanghai Enzyme Biotech Co., Ltd.
- Liver function indexes: Total bilirubin (TBIL), albumin (ALB), and international normalized ratio (INR) levels were measured in the serum of 4-ml fasting venous blood. ALB was detected by an enzymatic method using the reagent kit purchased from Shanghai Enzyme Biotech Co., Ltd.; TBIL was measured using a biochemical analyzer (Shenzhen Paier Biotechnology Co., Ltd., specification: PR-7450).

### Statistical Analysis:

All data were entered into an Excel spreadsheet and analyzed using SPSS software (version 26.0; IBM Corp, Armonk, NY, USA). The measurement data were represented as mean ± standard deviation. Independent sample t-test was used for inter-group comparison, and paired t-test was used for intragroup comparisons before and after comparison. Data were counted using the chi-square test to represent the number of use cases. *P*<0.05 was considered statistically significant.

## RESULTS

Clinical records of 60 patients (33 males and 28 females) were included in this study. Age ranged from 28 to 78 years, with an average of 53.92 ± 10.40 years. There were 31 patients in the TIPS group and 29 in the SST group, with no significant difference in baseline data between the two groups (*P*>0.05) (Table-I). The blood transfusion volume, hemostasis time, and hospital stay were significantly higher in the TIPS group than in the SST group (*P*<0.05) (Table-II).

**Table-I:** Comparison of baseline data between two groups.

Baseline data	TIPS group (n=31)	SST group (n=29)	χ^2^/t	P
Gender (male/female)	20/11	13/16	2.347	0.126
Age (year)	53.26±10.98	54.62±9.89	-0.504	0.616
Child Pugh grading of liver function			1.181	0.554
A	15 (48.39)	18 (62.07)		
B	11 (35.48)	8 (27.59)		
C	5 (16.13)	3 (10.34)		
Types of liver cirrhosis			7.164	0.127
Hepatitis B cirrhosis	16 (51.62)	16 (55.17)		
Hepatitis C cirrhosis	4 (12.90)	5 (17.24)		
Autoimmune liver disease and cirrhosis	4 (12.90)	5 (17.24)		
Alcoholic cirrhosis	6 (19.35)	0 (0.00)		
Other	1 (3.23)	3 (10.35)		

**Table-II:** Comparison of treatment outcomes between two groups.

Group	n	Blood transfusion volume (u)	Hemostasis time (hour)	Hospital stay (day)
TIPS group	31	3.13±1.18	23.13±4.90	14.68±5.80
SST group	29	4.41±1.15	27.45±6.00	22.17±5.17
*t*		-4.274	-3.064	-5.268
*P*		<0.001	0.003	<0.001

Before the treatment, PVD, PVV, PVP, and PVF were comparable in the two groups (*P*>0.05). After the treatment, these indexes decreased in both groups compared to pretreatment levels and were significantly lower in the TIPS group than in the SST group (*P*<0.05) (Table-III). Before the treatment, ACF, MI, and ACA levels were similar in the two groups (*P*>0.05). Conversely, after the treatment, all groups demonstrated a significant increase in levels of these indicators. Post-treatment levels of ACF, MI, and ACA in patients who underwent the TIPS procedure were significantly higher than in the SST group (*P*<0.05) (Table-IV).

**Table-III:** Comparison of hemodynamic status between two groups before and after treatment.

Item	Group	n	PVD (mm)	PVV (cm/s)	PVP (mmHg)	PVF (ml/minute)
Before treatment	TIPS group	31	14.51±1.80	22.32±1.70	28.64±2.45	860.90±180.45
	SST group	29	15.08±1.87	21.58±2.30	27.58±2.04	835.06±169.47
	*t*		-1.193	1.414	1.808	0.571
	*P*		0.238	0.163	0.076	0.570
After treatment	TIPS group	31	11.02±1.82a	18.06±1.74a	23.32±2.31a	536.65±82.06a
	SST group	29	13.00±1.74a	20.24±2.29a	25.93±2.61a	610.34±156.31a
	*t*		-4.292	-4.149	-4.095	-2.264
	*P*		<0.001	<0.001	<0.001	0.029

*Note:* Compared to before treatment in this group, ^a^*P* < 0.05; portal vein diameter (PVD), portal vein velocity (PVV), portal vein pressure (PVP), portal vein flow (PVF).

**Table-IV:** Comparison of gastric motility index levels before and after treatment between two groups.

Item	Group	n	ACF (time/hour)	MI	ACA (mmHg)
Before treatment	TIPS group	31	40.22±4.98	9.70±1.63	21.67±2.48
	SST group	29	41.31±5.73	10.24±1.92	22.24±2.29
	*t*		-0.783	-1.156	-0.912
	*P*		0.437	0.252	0.365
After treatment	TIPS group	31	59.35±6.56a	13.09±1.81a	44.22±5.60a
	SST group	29	51.89±5.97a	11.55±2.24a	38.48±4.66a
	*t*		4.591	2.941	4.297
	*P*		<0.001	0.005	<0.001

*Note:* Compared with before treatment in this group, ^a^*P*<0.05; antral contraction frequency (ACF); motility index (MI); and anstral contraction amplitude (ACA).

Similarly, pretreatment levels of ET, NO, and VEGF were comparable in both groups (*P*>0.05). After the treatment, the levels of ET, NO, and VEGF in both groups were significantly reduced compared to before treatment and were markedly lower in the TIPS compared to the SST group (*P*<0.05) (Table-V). Table-VI.

**Table-V:** Comparison of vascular endothelial function between two groups before and after treatment.

Item	Group	n	ET (pg/ml)	NO (umol/L)	VEGF (pg/ml)
Before treatment	TIPS group	31	109.35±36.44	94.74±13.25	109.16±24.34
	SST group	29	107.51±31.43	92.13±16.49	111.58±26.59
	*t*		0.208	0.676	-0.369
	*P*		0.836	0.502	0.714
After treatment	TIPS group	31	83.03±15.69a	63.48±9.50a	81.12±12.48a
	SST group	29	94.75±17.60a	74.89±11.68a	92.31±16.72a
	*t*		-2.728	-4.163	-2.919
	*P*		0.008	<0.001	0.005

*Note:* Compared with before treatment in this group, ^a^*P*<0.05; endothelin (ET); nitric oxide (NO); and vascular endothelial growth factor (VEGF).

**Table-VI:** Comparison of TBIL, ALB, and INR before and after treatment between two groups.

Item	Group	n	TBIL (umol/L)	ALB (g/L)	INR
Before treatment	TIPS group	31	23.35±3.32	41.45±6.62	2.13±0.60
	SST group	29	24.48±4.13	42.37±7.93	2.31±0.70
	t		-1.168	-0.493	-1.054
	P		0.248	0.624	0.296
After treatment	TIPS group	31	22.38±3.90	41.06±6.49	2.09±0.60
	SST group	29	21.96±4.20	40.51±7.11	2.04±0.56
	t		0.403	0.311	0.343
	P		0.689	0.757	0.733

*Note:* total bilirubin (TBIL); Albumin (ALB); international standardized ratio (INR)

As shown in Table-VI, there were no significant changes in the pretreatment and post-treatment levels of TBIL, ALB, and INR between the two groups (*P*>0.05). After six months of follow-up, the incidence of rebleeding and death in the TIPS group was significantly lower than that in the SST group (*P*<0.05) (Table-VII).

**Table-VII:** Comparison of rebleeding and mortality between two groups.

Group	N	Rebleeding	Death
TIPS group	31	4 (12.90)	2 (6.45)
SST group	29	11 (35.48)	8 (27.59)
*χ^2^*		5.006	4.819
*P*		0.025	0.028

## DISCUSSION

This study demonstrated the effectiveness and feasibility of the TIPS procedure for treating patients with liver cirrhosis complicated by EVB. Our results showed that compared to the conventional treatment, TIPS can effectively improve patients’ hemodynamic status, restore gastrointestinal function, and reduce the rates of rebleeding and mortality, ensuring a satisfactory prognosis. TIPS is a portosystemic shunt surgery that establishes a channel between the portal and hepatic veins, significantly reducing portal vein pressure and improving hemodynamics. Therefore, early TIPS treatment is associated with better hemostatic and hypotensive effects.^7^-^9^ The study by He et al.^7^ showed that TIPS treatment for liver cirrhosis complicated with EVB can effectively stop bleeding, with a mortality rate of only 3.60%. This finding is consistent with the results of the present study. Wang et al.^8^ used TIPS to treat patients with recurrent portal hypertension and variceal bleeding who had previously undergone open splenectomy and esophagogastric disconnection. Their results showed that the hemostasis rate of patients who underwent TIPS was as high as 97.37%, and both PVP and portal vein pressure gradient significantly decreased.

The trend of changes in the hemodynamically-related indicators observed in our study was consistent with previous research. During the TIPS procedure, a constructed shunt between the portal vein and hepatic vein allows diverting portal blood flow to the inferior vena cava, thereby reducing portal vein pressure.^9^ During the procedure, synchronous embolization is performed on the esophageal and gastric veins to block the flow of portal vein blood to the esophageal and gastric veins, effectively controlling bleeding that is caused by varicose vein rupture, regulating hemodynamic status, and improving body function.

Several studies have explored the application value of TIPS in the treatment of liver cirrhosis complicated by EVB.^10^,^11^ Yin Xiaochun et al.^10^ used TIPS to treat patients with EVB and portal vein cavernous transformation and demonstrated a significant decrease in portal vein pressure, with a cumulative stent patency rate of 86.8% during the one-year follow-up period. The incidence rates of upper gastrointestinal bleeding and hepatic encephalopathy were 10.0% and 22.2%, respectively, and the survival rate was as high as 88.9%.

Li Peijie et al.^11^ used TIPS surgery and drug-combined endoscopy to treat patients with liver cirrhosis complicated with EVB and showed that the incidence of recurrent bleeding in the TIPS treatment group was considerably lower at six weeks, one year, two years, and five years after the surgery compared to patients who received drug-combined endoscopic treatment. Together with our observations, these results further confirm that TIPS surgery can effectively reduce the rate of recurrent bleeding in the short, medium, and long term and suggest that early implementation of TIPS treatment can maximize the survival benefits of patients.

The impact of changes in endothelial factors, such as VEGF, endothelin and NO, on liver cirrhosis and portal hypertension has been a focus of extensive research. A study by Zhan Hongjing et al.^12^ has shown that VEGF can promote the growth of endothelial cells and accelerate angiogenesis. As VEGF expression increases with the progression of liver cirrhosis, VEGF-promoted endothelial growth and vascular permeability lead to splenic vascular proliferation, increased blood storage, and increased splenic pressure, thereby exacerbating portal hypertension.^13^ NO exerts multiple pathological and physiological effects.

Elevated levels of NO in patients with liver cirrhosis can cause arterial dilation, leading to a state of high arterial pressure circulation, affecting vasoconstriction and damaging the integrity of the intestinal epithelium.^14^ ET is an active peptide with a vasoconstrictive function whose expression increases with the progression of liver cirrhosis and portal hypertension.^15^ This, in turn, can promote abnormal vasoconstriction of the portal vein system, increase reflux resistance, and portal hypertension.^16^ In our study, the ET, NO, and VEGF levels in the TIPS group were lower than those in the SST group. Our results suggest that TIPS can effectively downregulate the expression of ET, NO, and VEGF, improve endothelial function, and ensure good disease outcomes. It is plausible that the TIPS procedure can reduce the blood flow and pressure of the portal vein and collateral circulation, thus reducing the pressure of esophageal and gastric varicose veins, regulating the hemodynamic status of esophageal veins, correcting hemodynamic disorders, reducing blood reflux resistance, and preventing high arterial pressure circulation.^12^,^14^,^16^-^18^

In addition, this study found no significant difference in the liver function of patients in both groups, as reflected by comparable levels of TBIL, ALB, and INR before and after the treatment. Our results indicate that the TIPS procedure for liver cirrhosis complicated by EVB is safe and is not associated with damage to liver function. Our results contradict a previous report by Rajesh et al.^19^ that TIPS can cause reversible damage to liver function in patients with liver cirrhosis complicated by EVB. We may speculate that this discrepancy may be due to the variability in the patient’s basic health status, the severity of the condition, and operator-related skills.

Rajesh et al.^19^ emphasized the transient nature of the TIPS-induced decrease in liver function. They suggested that the reason for the observed TIPS-induced liver injury is related to the construction of an artificial shunt channel between the portal vein and hepatic vein, which diverts blood flow to the inferior vena cava, reducing liver blood flow, causing insufficient oxygen supply, and leading to varying degrees of liver injury. However, over time, the compensatory function of the liver and the body’s adaptability to liver damage increase, and the proper function is restored. Moreover, due to the buffering effect of the hepatic artery, the metabolism of blood flow perfusion in the liver tissue increases, liver blood flow perfusion gradually returns to normal, and liver function gradually recovers.

Patients undergoing EVB are still at risk of recurrence and rebleeding after hemostasis. The results of the six months follow-up in our study showed that the incidence of recurrent bleeding and mortality in the TIPS group was significantly lower than that in the SST group. Our results suggest that TIPS has better therapeutic effects, can stop bleeding, and reduces mortality, which is consistent with the report of Dai Huan et al.^20^ Studies have shown that TIPS effectively reduces portal vein pressure through the shunt, improves gastroesophageal varices, reduces rebleeding risk, and has medium-to-long-term therapeutic effects.^21^,^22^

The current study aimed to fill the gap in the current lack of research on the clinical efficacy of TIPS in treating EVB of liver cirrhosis patients, and it is shown that TIPS treatment is associated with improved hemodynamic status, gastric motility, vascular endothelial function, and lower incidence of rebleeding and mortality, which can provide evidence for clinical practice.

### Limitations:

First, this is a single-center retrospective study. Second, the postoperative follow-up period was only six months. Longer follow-up times are required to verify our results. Finally, the impact of the two methods on the long-term functional recovery of patients was not analyzed. Further high-quality research is needed to verify the therapeutic effects of TIPS.

## CONCLUSION

In patients with liver cirrhosis complicated by EVB, TIPS treatment does not affect liver function and is associated with improved hemodynamic status, gastric motility, and vascular endothelial function, as well as lower incidence of rebleeding and mortality compared to conventional drug therapy.

### Authors’ Contributions:

**QZ and Qiong Zi:** Study design, literature search and manuscript writing.

**GD, YL and YZ:** Data collection, data analysis, interpretation and critical review.

**QZ and Qiong Zi:** Manuscript revision and validation, critical analysis.

All authors have read, approved the final manuscript and are responsible for the integrity of the study.
